# Orthographic and Phonological Priming Effects in the Same–Different Task

**DOI:** 10.1037/xhp0000548

**Published:** 2018-10-11

**Authors:** Sachiko Kinoshita, Michael Gayed, Dennis Norris

**Affiliations:** 1 Department of Psychology and ARC Centre of Excellence in Cognition and its Disorders (CCD), Macquarie University; 2 Department of Psychology, Macquarie University; 3 MRC Cognition and Brain Sciences Unit, Cambridge, United Kingdom

**Keywords:** orthographic code, phonological priming, masked priming, same–different task, writing systems

## Abstract

Masked priming tasks have been used widely to study early orthographic processes—the coding of letter position and letter identity. Recently, using masked priming in the same–different task Lupker, Nakayama, and Perea (2015a) reported finding a phonological priming effect with primes presented in Japanese Katakana, and English target words presented in the Roman alphabet, and based on this finding, suggested that previously reported effects in the same–different task in the literature could be based on phonology rather than orthography. In this article, the authors explain why the design of Lupker et al.’s experiment does not address this question; they then report 2 new experiments that do. The results indicate that the priming produced by orthographically similar primes in the same–different task for letter strings presented in the Roman alphabet is almost exclusively orthographic in origin, and phonology makes little contribution. The authors offer an explanation for why phonological priming was observed when the prime and target are presented in different scripts but not when they are presented in the same script.

## Public Significance Statement

The masked priming same–different task, in which people are asked to decide whether the target (e.g., CULT) preceded by a briefly presented prime (cult) is the same or different from the referent (cult) is used widely to study early orthographic processes with letter and word stimuli. A recent study, using primes written in a different writing system from the referent and target found phonological priming effects and, based on this result, suggested that the masked priming effects from this task could be phonological, rather than orthographic. The article explains why that finding does not address the issue, and provides empirical evidence against the suggested possibility. Understanding the processes underlying experimental tasks is important for meaningful interpretation of data.

In visual word recognition research, the past decade has seen much progress in understanding the “front-end”—early orthographic processes involved in the coding of letter identity and letter position. Much of this research has made use of masked priming. Priming tasks have the merit that they can be used to map out the similarity between the representations developed at different points in word recognition. For example, a commonly used masked priming paradigm pioneered by Forster and Davis (1984) uses the lexical-decision task. A prime is presented briefly (typically for no more than 50 ms) following a forward mask (e.g., #####) and is backward-masked by the target to which a response is required. The participant’s task is to decide whether the target is a word or not. A rich body of benchmark data on early orthographic processes have been reported using the masked priming procedure. For example, masked priming has been used to investigate the representation of letter order in reading by using primes in which letters in the target have been transposed (e.g., jugde–JUDGE). Transposed-latter (TL) priming facilitates the recognition of the target word almost as much as the identity prime, and more than a “substituted (or ‘replaced’) letter” prime in which the corresponding letters are replaced by unrelated letters (e.g., junpe–JUDGE; e.g., Forster, Davis, Schoknecht, & Carter, 1987; Perea & Lupker, 2003, 2004). One limitation of using masked priming with the lexical-decision task is that priming effects are generally only observed for word targets (Forster, 1998). This makes it difficult to disentangle lower-level orthographic effects from higher-level lexical effects. Norris and Kinoshita (2008) introduced a variant on the masked priming paradigm where the requirement to perform lexical decision on the target was replaced by the task of deciding whether the target is the same as, or different to, a referent stimulus presented immediately (usually about 1 s) before the prime and target. In this same–different masked priming task, priming is found for both words and nonwords when the target is the same as the referent, but for neither words or nonwords when the target is different. Norris and Kinoshita (2008; see also, e.g., Kinoshita & Norris, 2009; Norris, Kinoshita, & van Casteren, 2010) put forward an account of masked priming based on the Bayesian reader model of visual word recognition developed by Norris (2006, 2009). According to the Bayesian reader, perception involves Bayesian inference based on accumulation of noisy evidence, with the hypothesis for which evidence is accumulated being determined by the goal of the task. A critical assumption of the theory is that, under the circumstances of masked priming, the prime and the target are processed as a single perceptual object. Evidence from both the prime and the target continuously updates the probability of the hypotheses required to perform the task. Norris and Kinoshita (2008) explained that from this perspective, masked priming in the same–different task is just like that in lexical decision, except that the referent consists of a single string of letters maintained in short term memory, whereas in lexical decision, it is the whole lexicon in lexical memory.

Using the same–different task, Kinoshita and Norris (2009) showed for the first time robust TL priming effects are observed with pseudowords (e.g., nisdt–NIDST), indicating that TL priming effects are not lexical in origin. García-Orza, Perea, and Munoz (2010) replicated this finding, and extended the finding of TL priming effects to consonant strings, digit strings, and nonalphanumeric symbol strings (e.g., + > &%). García-Orza, Perea, and Estudillo (2011) further extended the finding of TL priming effects to a sequence of simple geometrical shapes.^1^ These and related findings (e.g., Norris et al., 2010) provided a body of evidence for the “noisy position” interpretation of TL priming effects, namely, that TL priming effects reflect the ambiguity in the coding of letter position within a string, which originates in noisy perception of spatial position—the core assumption of the overlap model proposed by Gómez, Ratcliff, & Perea (2008).^2^ (For an alternative view that TL priming effects reflect the role of specialized orthographic representations such as “open bigrams” (ordered letter pairs), see, e.g., Grainger & van Heuven, 2003; Grainger, Granier, Farioli, van Assche, & van Heuven, 2006; Whitney, 2001. For evidence against open bigrams, see, for example, Kinoshita & Norris, 2013).

The masked priming same–different task has also been used to investigate whether reading is mediated by abstract letter identities–letter representations that are invariant to variations in font, size, and case. Previous studies (e.g., Arguin & Bub, 1995; Bowers, Vigliocco, & Haan, 1998; Ziegler, Ferrand, Jacobs, Rey, & Grainger, 2000) sought evidence for the involvement of abstract letter identities by testing whether priming effects are found independent of visual similarity between uppercase and lowercase letter pairs (e.g., c/C and x/X are visually similar; a/A and r/R are visually dissimilar). These studies used the alphabet decision task (where the foils are nonalphabetic symbols like % and #) and have not found evidence that the task uses abstract letter identities (see Grainger, Rey, & Dufau, 2008 for a summary). However, priming in this task is generally weak, and the interaction (or lack thereof) with visual similarity is therefore difficult to interpret. As noted by Arguin and Bub (1995), the alphabet decision task does not necessarily require unique letter identification; instead a positive alphabetic decision response can be generated on the basis of “global letter activity,” defined as the summed activation across all letter representations, rather than on the basis of a specific abstract letter representation.^3^ Noting this limitation, Kinoshita and Kaplan (2008) instead used the cross-case letter match task in which the participant’s task is to decide whether the target (e.g., A) is the same letter as the referent presented in the opposite case (e.g., a). They (see also Norris & Kinoshita, 2008) found equally robust priming effects for visually dissimilar prime-target letter pairs and visually similar letter pairs, which was taken as the first clear evidence for priming of abstract letter identities. Carreiras, Perea, and Abu Mallouh (2012) used this task to study whether the abstract letter codes support letter recognition in Arabic, which has extensive position-dependent allography (the same letter may take up to four different shapes depending on its position within the letter string). Consistent with the results found with the Roman alphabet, they found robust priming effects for letter pairs differing in shape, which they took to suggest that “priming of abstract letter representations may be universal” (the title of their paper). Carreiras, Perea, Gil-López, Abu Mallouh, and Salillas (2013) combined the event related potentials methodology with the masked priming same–different task to study the neural signatures of abstract letter identities in readers of English and Arabic.

The conclusion that can be drawn from this body of work is that masked priming effects in both lexical decision and the same–different task inform us about the role of orthographic representations at the level of abstract letter identities. However, recently, Lupker et al. (2015a) reported a finding (to be described shortly) using the same–different task which, they suggested, implies that previously reported priming effects with the Roman alphabet may have been due to phonological, rather than orthographic, similarity. If true, this poses a serious challenge to the previous interpretations that have come to be well-accepted—for example, that TL similarity effects are orthographic—rather than phonological (e.g., Grainger, 2008; Perea & Carreiras, 2006); also, as noted above, the priming effects observed in the letter match task are interpreted as evidence for abstract letter identities, which are orthographic, not phonological representations. Should the field in general revise its views on whether the masked priming findings reported to date really were due to phonological, rather than orthographic, similarity?

In this article, we explain why the claim that the masked priming effects in the same–different task using orthographically similar prime and target pairs written in the alphabetic script may have reflected phonology does not necessarily follow from their finding. We then report two new experiments to empirically test the claim and find little evidence.

Lupker et al. (2015a) conducted two experiments, both using the same–different task with Japanese-English bilinguals. The critical feature of their experiments is that the referent and target were English words presented in the *Roman alphabet* (e.g., referent–south; target–SOUTH), and the masked prime was a transliteration of the target word presented in the *Japanese Katakana* script (e.g., サウス,/sa.u.su/), which the authors referred to as the cognate prime. Relative to the unrelated control prime (another word presented in Katakana which was a transliteration of an unrelated word, e.g., カーブ,/ka.R.bu/, “curve”), the cognate prime facilitated the SAME response. In their Experiment 2, instead of cognate primes, the authors used “phonologically similar primes” which were pseudowords generated by substituting one Katakana (which also changed one phoneme) in the cognate prime, for example, サウス (/sa.u.su/) > サオス (/sa.o.su/). The phonologically similar primes also produced facilitation relative to the control primes. Lupker et al. correctly noted that as the Roman alphabet and the Katakana share no letters in common any priming observed here cannot be orthographic in origin. Taking the results of Experiment 1 and 2 together, the authors concluded that the priming effect observed here most likely reflected phonological overlap between the prime and target.

We have no disagreement with this interpretation of their data. Our concern is that these data are silent with regards the locus of priming when primes and targets are presented in the same orthography; more specifically, when they share a common representation at the level of abstract letter identities. Lupker et al. (2015a) presented the prime and target in different writing systems that share no letters in common (Japanese Katakana and the Roman alphabet), which, by definition have no orthographic overlap. Therefore, in their task, there could not possibly be any orthographic priming. Any priming observed must be phonological. However, this finding by itself does not tell us about the locus of priming when there is orthographic overlap between the prime and target.

Writing systems like the Roman alphabet and the Japanese kana are designed to code phonology. For example, in writing systems using the Roman alphabet, letters often map onto a single phoneme (e.g., in English, *d* maps onto /d/); in the Japanese kana writing system a kana maps onto a mora (a syllable-like unit consisting of a single vowel, or a consonant-vowel combination, e.g., サ maps onto /sa/). Within such a writing system, stimuli that share letter/character sequences at the abstract letter identity level and hence are orthographically similar are also phonologically similar: for example, score and SCORE are orthographically and phonologically identical. When the prime and target are presented in the same writing system and have orthographic overlap, it is therefore difficult to avoid phonological overlap.

Noting this systematic relationship between orthographic and phonological similarity within the alphabetic writing system, Kinoshita and Norris (2009, Experiment 3) specifically tested whether the orthographic priming effects in the same–different task are due to phonology. In that experiment, three types of primes were used, manipulating orthographic and phonological overlap. The prime either shared the abstract letter identities and hence also phonology with the target (identity prime, e.g., score–SCORE), only phonology (pseudohomophone/PSH prime, e.g., skore–SCORE) or did not share orthography or phonology (control prime, e.g., smore-SCORE). The PSH prime and the control prime were matched on the degree of orthographic overlap with the target as they both differed from the target in exactly one letter (always a consonant), in the same position (second, third, or fourth letter of a five-letter word). Kinoshita and Norris (2009) reasoned that “if priming is phonological, we would expect both the identity prime and pseudohomophone prime conditions to facilitate “same” responses relative to the control prime condition. On the other hand, if priming in this task is purely orthographic, based on abstract letter identities, we would expect the pseudohomophone and control prime conditions not to differ and the identity prime condition to be faster than the other two prime conditions” (p. 8). The results supported the latter prediction: The identity primes produced (26 ms) facilitation relative to the PSH primes, but there was little difference (2 ms) between the PSH and control prime conditions, indicating that priming was due entirely to orthographic overlap, and phonological overlap made little contribution. On the basis of this finding Kinoshita and Norris (2009) concluded that phonology makes little contribution to orthographic priming effects in the same–different task.

Lupker et al. (2015a) argued that phonological priming effect was absent in that study because the phonological manipulation was weak, as the PSHs and their control nonwords differed in only one phoneme, and that their own study produced a sizable phonological priming effect because their phonological manipulation (comparing primes in Japanese katakana that were transliteration of the English word target (e.g., サウス,/sa.u.su/–SOUTH) with primes that had no phonological overlap with the target (e.g., カーブ,/ka.R.bu/- SOUTH) was stronger. However, the strength of phonological manipulation, or the size of the phonological priming effect, is not the issue. The issue at debate is whether the priming produced by the prime-target pairs that share abstract letter identities and hence are orthographically (and consequently also phonologically) similar could instead be explained in terms of phonology. What Lupker et al. (2015a) investigated is different: Whether two stimuli that have no orthographic overlap—clearly, Japanese katakana and the Roman alphabet do not share abstract letter identities—could produce priming based on shared phonology. However large the observed phonological priming effect might be, this manipulation does not address the question whether the priming produced by an orthographically similar prime was instead due to phonological similarity. To answer that question, the orthographic priming effect needs to be compared to the phonological priming effect within the same writing system. Note also that the criticism that a manipulation of a single phoneme is weak is difficult to sustain given that, in Kinoshita and Norris’ (2009) experiment, the orthographic manipulation (score-SCORE vs. skore-SCORE) that produced a substantial (26 ms) priming effect also involved a difference of only one letter. That is, both the orthographic and phonological manipulations involved changing the same proportion of the identity prime to form either the PSH prime or the orthographic control prime. Note further that the claim that manipulation of a single phoneme is weak is contradicted by the fact that the masked onset priming manipulation involves a single letter/phoneme (e.g., bark–BENCH vs. dark–BENCH) and it produces a robust effect in naming (e.g., Forster & Davis, 1991; Kinoshita, 2000; Nakayama, Kinoshita, & Verdonschot, 2016).^4^ That is, single letter/phoneme manipulations can, and do, produce robust phonology-based effects where phonology is involved.

It is worth being quite explicit about the different predictions that follow from the assumptions that priming based on the abstract letter identities is mainly orthographic or mainly phonological. If priming in the same–different task is entirely phonological, this generates the following predictions: A PSH prime (e.g., skore–SCORE) will be phonologically identical to the target and should therefore produce exactly the same amount of priming as an orthographic identity prime (e.g., score–SCORE). That is, the PSH and identity conditions should be the same. In Kinoshita and Norris’s (2009) Experiment 3, they differed by 26 ms. An orthographic control prime (e.g., smore–SCORE) will differ from the PSH (and the target) by a single phoneme and hence produce less priming than the PSH prime. It did not: The PSH prime condition was merely 2 ms faster than the orthographic control prime condition.

On the other hand, if priming is orthographic, based on shared abstract letter identities, an orthographic identity prime (e.g., score–SCORE) is orthographically identical to the target, and an orthographic control prime that differs from the target by a single letter (e.g., smore–SCORE) should produce less priming. The PSH prime (e.g., skore–SCORE) also differs by a single letter and should therefore produce the same amount of priming as the orthographic control prime. In Kinoshita and Norris’s (2009) Experiment 3, these two conditions differed by only 2 ms and were both slower than the identity prime condition. In sum, the pattern of results in Kinoshita and Norris’s (2009) Experiment 3 clearly indicated that phonological overlap between the prime and target makes almost no contribution to the priming produced by the prime that is orthographically and phonologically identical to the target, and this (together with the results of Experiments 1 and 2) is what led them to conclude that “the same–different task holds considerable promise as a tool for examining the nature of prelexical orthographic representations” (p. 13).

Nevertheless, Kinoshita and Norris’ (2009) experiment stands as the only one to date that examined the contribution of phonology to orthographic priming effects in the same–different task.^5^ We felt therefore that it was important to replicate this result, and also to quantify the amount of evidence using Bayes factors. The Bayes factor indexes the relative strength of evidence for one hypothesis over another, and unlike the conventional significance testing, it can determine whether nonsignificant results count against a theory, or whether the data are just insensitive (see Dienes, 2014; Rouder, Speckman, Sun, Morey, & Iverson, 2009). In addition, we quantifed the evidence for the relative contribution of orthography and phonology using the Bayes factor. Specifically, the likelihood that the orthographic priming effect is bigger than the phonological priming effect can be given by the ratio of the orthographic identity priming to the PSH priming: (PSH–Identity)/(1LD–PSH).

We began by performing a Bayes factor analysis of Experiment 3 of Kinoshita and Norris (2009). We used the same linear mixed effect model (Baayen, 2008; Baayen, Davidson, & Bates, 2008) with lme4 1.1–13 (Bates, Maechler, Bolker, & Walker, 2017), and lmerTest (Kuznetsova, Brockhoff, & Christensen, 2016) implemented in R 3.4.0 (2017–4-21, R Development Core Team, 2017), combined with the R package BayesFactor (v. 0.9.12–2). In that experiment, the Bayes factor was 75 in favor of the difference between the identity and PSH prime (26 ms), and 10 in favor of the null difference between the PSH and control prime conditions (2 ms). The ratio of the two Bayes factors indicating the likelihood of a larger orthographic priming effect relative to the phonological priming effect, was 750 (75/0.1).

We also computed the power to detect an effect as large as the orthographic identity priming effect observed in that experiment (.95, using the R package simr v. 1.03, Green & MacLeod, 2016). As discussed above, if phonology were to play as important a role in the same–different judgments as orthographic information, phonological priming should be as large as orthographic priming, so that is the size of effect we need to be able to detect. That experiment had 24 participants. The two experiments to be reported here increase the power by using 30 participants. Note that this estimate of power applies to the orthographic priming effect using null hypothesis significance testing. There are no established procedures for computing power for the Bayes factor analysis. Indeed, Dienes (2014) has argued that Bayes factors obviate the need to perform power analyses.

In the present study, we apply the same experimental manipulation to a new set of words (Experiment 1) and extend it to pseudoword stimuli (Experiment 2). In addition, we changed the position of the manipulated letter. In Kinoshita and Norris’s (2009) Experiment 3, the critical letter occurred in the second, third, and fourth position of five-letter words. It is arguable that the (orthographic as well as phonological) priming manipulation was not as strong as it could have been in that experiment because the critical letter was word-internal. Perceptual identification of a letter within a string is generally worse for internal positions and best for the initial position (see, e.g., Aschenbrenner, Balota, Weigand, Scaltritti, & Besner, 2017), and in the masked onset priming manipulation (which has been shown to produce phonological priming effects in tasks involving phonology) it is always the first letter that is manipulated. In the present experiments, we therefore manipulated the first letter (e.g., cult/kult/nult/CULT). In addition, we quantified the amount of evidence for the relative contribution of orthography and phonology using the Bayes factor.

## Experiment 1 (Words)

### Method

#### Participants

Thirty students (25 female, five male) from Macquarie University participated in the experiment in return for course credit. They were between the ages of 19 to 59 (mean 24.2).

#### Design

The experiment used the masked priming same–different task, and involved the factor prime type (identity, PSH, and control) manipulated within subjects. The dependent variables were response latency and error rate.

#### Materials

The critical stimuli were 60 four- and five-letter words. They were selected to have a simple consonant onset, containing the letters C, S, G, J or K, so that a PSH can be generated by substituting the initial letter. Examples are CULT (kult), SELF (celf), GYPSY (jypsy).

The words ranged in frequency (.17 to 709, mean 73.06 per million based on Celex frequency). The number of orthographic neighbors (as defined by the *N* metric, Coltheart, Davelaar, Jonasson, & Besner, 1977) ranged between 0 and 24, mean 10.27. Position-dependent bigram frequency ranged from 10 to 70.5, mean 61.0. All measures were based on the MC-Word database (Medler & Binder, 2005).

Each target word was paired with three types of prime: identity, PSH, and control. The *identity prime* was the word itself. The *PSH prime* was a pseudoword generated by substituting the initial letter with another letter that did not change the pronunciation (e.g., CULT -> kult). The *control prime* was a pseudoword generated by substituting the initial letter with another letter that changed the pronunciation (e.g., CULT > nult). Three list versions were constructed for the purpose of counterbalancing the assignment of primes to the three prime conditions. The 60 target words were divided into three sets matched on the mean number of letters and mean frequency, and in each list a target word was paired with one type of prime (identity, PSH, or control). The critical target words and primes are listed in the appendix.

Each target word was presented with a referent. In the same trials, the referent was the target word itself (e.g., referent–cult, target–CULT). In the different trials, the referent was another target word of the same length that shared as few letters as possible (e.g., referent–seed, target–CULT; referent–class, target–GYPSY). In 15 out of 60 cases where this was not possible the referent was not another target word but a word selected according to the same criteria (four- or five-letter word that contained as the initial letter C, S, G, J or K).

In addition to the critical stimuli, there were 12 practice items, selected according to the same criteria as the test stimuli. These items were not included in the analysis.

#### Apparatus and procedure

Participants were tested either individually or in pairs, seated approximately 60 cm in front of a flat screen monitor, upon which stimuli were presented. Each participant completed 120 test trials consisting of 60 same and 60 different trials, presented in one block. A different random order of trials was generated for each participant. The test trials were preceded by 12 practice trials containing a representative number of conditions.

Participants were instructed at the outset of the experiment that on each trial they would be presented with a word in lowercase letters (referent) above # signs, and their task was to decide whether a subsequently presented word, in uppercase letters, was the same of different from the referent (ignoring the difference in case). No mention was made of the presence of primes. Participants were instructed to press a key on a response pad marked “+” for same and a key marked “−” for different responses.

Stimulus presentation and data collection were achieved through the use of the DMDX display system developed by K. I. Forster and J. C. Forster at the University of Arizona (Forster & Forster, 2003). Stimulus display was synchronized to the screen refresh rate (10.01 ms).

Each trial started with the presentation of a referent word in lowercase letters, above a forward mask consisting of four or five # signs for 1,000 ms, in the center of the screen. The forward mask was replaced by the prime in lowercase letters presented for 50 ms, then by the target presented in uppercase letters for a maximum of 2,000 ms, or until the participant’s response. All stimuli were presented in Courier New size 10 font. Participants were given a feedback (the message “Wrong response” presented on the screen) only when they made an error.

### Results and Discussion

The analyses were performed using linear mixed effects modeling (Baayen, 2008; Baayen et al., 2008) with lme4 1.1–13 (Bates et al., 2017), and lmerTest (Kuznetsova et al., 2016) packages implemented in R 3.4.0 (2017–4-21, R Development Core Team, 2017). The linear mixed-effects model we report used log-transformed reaction time (RT; logRT) of correct trials as the dependent variable (the log transformation was used to meet the distributional assumption of linear mixed effects model to approximate a normal distribution) and was created using a backward stepwise model selection procedure, with model comparison performed using chi-squared log-likelihood ratio tests with maximum likelihood. We opted for the simpler model when the model fit was not improved by greater model complexity.

The preliminary treatment of RT data for this analysis was as follows. First, we examined the shape of RT distribution for correct trials (a total of 1,713 observations) and applied the log transformation. We excluded trials with RTs shorter than 250 ms (1 data point). This cutoff was determined by inspecting the Q-Q plots of log-transformed RT.

The statistical model we tested included as a fixed factor, primetype, referenced to the PSH. Intercepts for subjects and items were included as crossed random effects (models that included subject and/or item slopes for the primetype factor did not improve the model fit). In R, the statistical model we report is: logRT ∼ prime type + (1 | subject) + (1 | target).

The mean RT for each prime condition is shown in Table 1.^6^

The statistical model showed that the identity prime condition was significantly faster than the PSH prime condition: *B* = −.073, *SE* = .013, *t* = −5.633, *p* < .0001, but the PSH prime condition and the control prime condition did not differ: *B* = .012, *SE* = .013, *t* = .937, *p* = .349. To quantify the amount of evidence, we computed the Bayes factor using the BayesFactor package (Version 0.9.12–2, Morey & Rouder, 2015). The Bayes factor was 207,609 in favor of the difference between the identity and PSH prime, and 0.1 in favor of their being a difference between the PSH and control prime conditions. Bayes factor is an odds ratio, with 1 indicating equal evidence for two alternative hypotheses. The Bayes factor of 0.1 in favor of there being a difference between the PSH and control prime condition can be expressed as a Bayes factor of 10 in favor of the null difference between the PSH and the control prime condition. Based on Jeffreys’s (1961) recommendation that odds greater than 3 be considered “some evidence,” odds greater than 10 be considered “strong evidence,” and odds greater than 30 be considered “very strong evidence” for one hypothesis over another, the observed Bayes factors indicate there was an overwhelming evidence for the role of orthographic overlap, and strong evidence *against* the contribution of phonology. We remind the readers that the Bayes factor of 10 in favor of the null phonological priming effect is based on the statistical model treating subjects and items as crossed random factors. This indicates that the null phonological priming effect was not due to the experiment lacking in sensitivity or power (if this were the case, the Bayes factor would have been closer to 1). We can also combine these Bayes factors to ask an additional question: What is the likelihood that the orthographic priming effect is bigger than the phonological priming effect? This is given by the ratio of the two Bayes factors, which is 2 × 10^6^ (207,609/0.1). Taken together with the overwhelming evidence for the orthographic priming effect, the Bayes factor indicates that the priming effect produced by orthographic overlap cannot be explained in terms of phonology.

In sum, the results of Experiment 1 using word targets completely replicated the pattern reported by Kinoshita and Norris (2009, Experiment 3), indicating that the masked priming in the same–different task is sensitive to orthographic overlap in just a single letter (cult–CULT < kult–CULT), but showed no sensitivity to phonological overlap (kult–CULT = nult–CULT). In Experiment 2, we apply the same experimental manipulation to pseudoword targets.

## Experiment 2 (Pseudowords)

### Method

#### Participants

Thirty-one students (23 female, 7 male) from Macquarie University, none of whom participated in Experiment 1, took part in Experiment 2 in return for course credit. They ranged in age between 19 to 24 (mean 20.1).

#### Design

The design was identical to Experiment 1.

#### Materials

The critical stimuli were 60 four- and five letter pseudowords. They were generated according to the same criteria as the word targets used in Experiment 1, namely, have a simple consonant onset, containing the letters C, S, G, J or K, so that a PSH can be generated by substituting the initial letter. Examples are CUST (kust), SERL (cerl), JERT (gert). The number of orthographic neighbors ranged between 0 and 19, mean 6.08, and position-dependent bigram frequency ranged from 3.3 to 76, mean = 26.6.

Each target pseudoword was paired with three types of primes, identity, PSH, and control, generated in the same way as for the word targets. The critical targets and primes are listed in the appendix.

#### Apparatus and procedure

The apparatus and procedure were identical to Experiment 1.

### Results and Discussion

The preliminary treatment of RT data, and analysis method were identical to Experiment 1. The application of the same cutoff procedures for excluding outliers (<250 ms, 10 data points) resulted in 1,714 data points. As in Experiment 1, the statistical model we report is: logRT ∼ prime type + (1 | subject) + (1 | target).

The mean RT for each prime condition is shown in Table 2.[Table-anchor tbl2]

The statistical model showed that as in Experiment 1, the identity prime condition was significantly faster than the PSH prime condition: *B* = −.043, *SE* = .0117, *t* = −3.704, *p* < .001. Unlike Experiment 1, the phonological priming effect (indexed by the difference between the PSH prime and control prime conditions) was significant, *B* = .029, *SE* = .0117, *t* = 2.489, *p* < .02. The Bayes factor was 69 in favor of the difference between the identity and PSH prime, and 2 in favor of the difference between the PSH and control prime conditions. According to Jeffrey’s classification, this Bayes factor is to be considered “barely worth a mention.” In other words, there was again overwhelming evidence for an orthographic priming effect and, although the phonological priming effect for pseudoword targets was significant according to null hypothesis significance testing, the Bayes factor indicated only weak evidence for the effect. As in Experiment 1 we can also combine the Bayes factors to assess the likelihood that the orthographic priming effect is larger than the phonological priming effect. This is given by the ratio of the two Bayes factors—34.5 (69/2), “very strong evidence.” Note that numerically the ratio of the Bayes factors is much larger than the ratio of the corresponding RT differences (which does not take into account of the different strength of evidence for the two priming effects).

It is interesting that although there was no hint of phonological priming in Experiment 1 using word targets, an effect emerged in Experiment 2 using pseudoword targets. The phonological priming effect (kust–CUST < nust–CUST) could be based on the phonological representation of the referent (cust) created via the application of print-to-sound mapping. This phonological representation would match that for the PSH to produce priming relative to the control prime. The same is also possible for word referents (e.g., cult), so why was not there any phonological priming with words? One possible explanation is that it is because the spelling of words, but not pseudowords, is represented in lexical memory. The knowledge of spelling (e.g., cult is spelled with c, not k) could help maintain the representation of the referent string in the visual short-term memory/graphemic buffer, and because of this there was less scope for the phonological representation to influence priming when the referent is a word and spelling is less ambiguous. We will return to this issue in the General Discussion.

## General Discussion

Evidence from the masked priming same–different task has been used to draw inferences about the nature of the orthographic processes underlying word recognition. Lupker et al. (2015a) raised the possibility that “prelexical phonology could be available early enough in processing to play almost as important a role in same–different judgments as prelexical orthographic information” (p. 1291), and cautioned that it would be unsafe to attribute priming effects observed in that task to orthography rather than phonology. They presented two lines of argument in support of this possibility. The first is that previous unsuccessful attempts to demonstrate phonological priming in the same–different task have used too weak a manipulation of phonological overlap. (We have presented rebuttal of this argument in the introduction.) Second, and more important, they showed that phonological priming can be obtained when the prime and target are presented in different languages that are written in different writing systems (English written in the Roman alphabet and Japanese written in katakana) and therefore have no orthographic overlap.

The fact that priming can be obtained between words presented in different scripts clearly demonstrates that it is possible to obtain phonological priming in the same–different task. However, this says little about the size of the contribution phonology will make to priming when the prime and target are presented in the same script, but in different cases—that is, when they overlap orthographically (share the abstract letter identities). That can only be determined by looking for phonological priming under conditions where the prime and target are written in the same script. This is what Kinoshita and Norris (2009, Experiment 3) did, and they found no evidence of phonological priming. In the experiments reported here we used what should have been a stronger manipulation of phonological overlap—overlap at onset. In Experiment 1 using word stimuli, we once again found no evidence of phonological priming. This time we performed a Bayesian analysis which indicated strong support for the null hypothesis that there was no phonological priming. This was also the case when we reanalyzed Experiment 3 of Kinoshita and Norris (2009). The only hint of a statistically reliable effect of phonological overlap was in Experiment 2 with pseudoword stimuli using null hypothesis significance testing. However, the question is not whether some evidence for phonological priming can be found under some circumstances, but whether it plays as important a role as orthographic priming when primes and targets are presented in the same script. Even in Experiment 2, the Bayes factor analysis showed that there was very strong evidence that the orthographic priming was greater than any phonological priming.

The limited size of phonological priming effects is also the conclusion drawn by a major review of phonological priming effects in visual word recognition. Rastle and Brysbaert (2006) noted that phonological priming effects in the extant literature are generally small in size, and in their own lexical decision experiments they found a modest (but statistically significant) 9–13 ms PSH priming effect using prime–target pairs that ranged in orthographic similarity (e.g., klip–CLIP: orthographically similar; yuice–USE: orthographically dissimilar).^7^

We should make clear that we are not questioning the empirical status of the phonological priming effect reported by Lupker et al. (2015a). The phonological priming effects they reported were substantial (35–38 ms for the transliteration prime in Experiment 1; 26 ms for the prime with less complete phonological overlap in Experiment 2). Having made the case that masked priming effects are predominantly orthographic when prime and target are in the same orthography, we are therefore left with something of a puzzle as to why such a large phonological priming effect should emerge when there is no orthographic overlap. One possibility is that, in the absence of shared orthography, there is no competition between mismatching letters in the prime and target, and that this is what allows phonological priming to emerge. More specifically, if priming is mediated by representations of abstract letter identities then, when the letters are presented in the same writing system, the different letter identities in the PSH prime and target (e.g., k and c in kult–CULT) mismatch and they will compete with each other. In contrast, when a prime and a target are presented in different writing systems, the mismatching components of the prime and target will not share a common representation of abstract letter identity and there will be no competition at the orthographic level. For example, サウス and SOUTH do not share the common abstract letter identities and will not compete with each other at the orthographic level. However, they share a common phonological representation, and phonological overlap will now be able to produce priming. In effect, primes in different scripts will eliminate the competition between abstract letter identities that normally prevents the emergence of phonological priming.

It is of interest to note that in the present Experiment 2 using pseudoword referents and targets (e.g., cust/CUST), unlike the experiments using word referents and targets, a small phonological priming effect emerged, and it was suggested that it may be because the “correct” spelling for pseudowords is not known (i.e., unlike words, e.g., cult, it is less clear that a pseudoword cust is spelled with c, not k). The emergence of phonological priming effect for pseudowords may have the same basis as the phonological priming effect observed with primes and targets written in different writing systems. That is, when the orthographic information is less certain (e.g., the referent cust may have contained the letter c or k) there is less scope for competition between the mismatching letters in the PSH prime, and this allows the phonological priming effects to emerge.

Similar effects of shared versus nonshared orthography have been reported elsewhere. For example, priming by translation equivalents (e.g., rey (king in Spanish)–KING) is weak when the prime and target are written in the same writing system (e.g., the Roman alphabet used to write Spanish and English words) but robust when they are presented in different writing systems like Hebrew and English (Gollan, Forster, & Frost, 1997, lexical decision) or Japanese katakana and English (Lupker, Nakayama, & Perea, 2015b, lexical decision and the same–different task). Exactly the same argument should apply to these translation priming results: The mismatching letters in rey and KING will compete orthographically, but if they are presented in different scripts (Hebrew and the Roman alphabet; Japanese katakana and the Roman alphabet) they will not compete with each other at the orthographic level. Translation equivalents do share a common semantic representation, and the semantic overlap will now be able to produce priming. Taken together these findings suggest that in both lexical decision and the same–different task, the orthographic relationship (within the same writing system) between the prime and target dominates the priming effect and prevents phonological and semantic priming effects from emerging; in contrast, when the prime is written in a different writing system from the target, there is no competition between the different orthographic representations and this allows these priming effects to emerge. This putative role of orthographic competition in modulating phonological (and translation/semantic) priming effects needs to be investigated in future. For now, the point we make is that the phonological priming effects with prime and targets within a single writing systems are different from those observed across different writing systems, and also that the finding of phonological priming effects across different writing systems does not entail that the priming produced by orthographically similar primes within the Roman alphabet system is phonological, and this is what our experiments demonstrated.

Lupker et al. (2015a) also argued that their finding calls into question the support provided by the findings of cross-case identity priming effects in the letter match task for the conclusion that letter matching and, hence, priming in the cross-case letter-matching task, is mainly based on abstract orthographic codes. In the cross-case letter match task, the referent and target are single letters in differing case (e.g., referent–a, target A). The key result reported in these studies is that the size of priming effect did not depend on the visual similarity of the prime-target letter pairs differing in case (e.g., c and C are visually similar; a and A are visually dissimilar). Lupker et al. correctly noted that this pattern of data is consistent with priming being based on either phonological codes or abstract orthographic codes. However, here again, Lupker et al.’s experiments do not speak to the nature of the code underlying the priming effect, because their experiments examined primes and targets in different scripts. There are two further problems with their argument. First, Lupker et al. (2015a) used word stimuli and drew implications for letter representations; that is, they adopted what Bowers et al. (1998) referred to as the “letter–word equivalence assumption,” an (unwarranted) assumption that the results obtained with word stimuli have implications for letters. Specifically, in the cross-case letter match (e.g., does “D” have the same letter identity as “d”), if the letter priming effects were phonological, they would be based on letter names (e.g., “dee”). The phonological priming effect found by Lupker et al. (2015a) between the words written in Japanese katakana (e.g., サウス,/sa.u.su/) and the Roman alphabet (e.g., SOUTH) could not have been based on letter names (e.g., “ess,” “o,” “u,” “tee,” “aitch”), and hence their finding has little to say about the nature of codes underlying the letter priming effects in the cross-case letter match task. Second, their characterization of the extant literature on sequential letter-matching task to support their claim that letter priming effects were phonological is selective, as we point out below.

Lupker et al. (2015a, p. 1291) quoted Proctor (1981, p. 302) as stating that “All sequential matches are apparently based on name codes” to make the case for the phonological basis of letter priming effects in the letter match task. However, this quote mischaracterizes Proctor’s position who says later in the same review paper “the identification and cognitive coding of a stimulus does not require that a name be involved” (p.321). Lupker et al. (2015a) also make no reference to the extensive discussion in Kinoshita and Kaplan (2008) of the debate in the 1970s and 80’s concerning whether the code used in the cross-case letter match is based on abstract perceptual code or a phonological code. Kinoshita and Kaplan (2008) explicitly noted a point made by Proctor (1981) that the “name code” cannot be the code supporting the match decisions for sequentially presented pairs, because participants were able to match nonalphanumeric characters for which a name is not available, such as Japanese letters with which the participants were unfamiliar. In summing up this literature, Kinoshita and Kaplan (2008) pointed out that “Although earlier work has assumed that the letter match task for cross-case letters (which was then called a name match or nominal match) is based on phonological representation of letter names, there is much evidence from later studies that rules out this possibility” (p. 1876), citing among others (e.g., Carrasco, Kinchla, & Figueroa, 1988; Rynard & Besner, 1987), Boles and Eveland (1983) who showed that phonological similarity of letter names (e.g., similar: A–J, B–C; dissimilar: A–C, B–J) had no impact on different decisions. The overwhelming consensus in the literature, including data from neuropsychology (Coltheart, 1981; Rynard & Besner, 1987) and neuroscience (e.g., Dehaene et al., 2004; Polk & Farah, 2002; Rothlein & Rapp, 2014), is that cross-case letter matches are based on a “non-phonological, case-independent, font-independent, abstract representation” (Bigsby, 1988, p.455). The present results indicating a minimal role of phonology in the masked priming of letter strings in the sequential match is thus entirely in line with the view that letter match is based on an abstract letter code, rather than a phonological code.

Consistent with this view, recently Kinoshita, Schubert, and Verdonschot (2018) showed that priming by allographs (letters which are different in form but share the same abstract letter identity, like the uppercase and lowercase letters of the Roman alphabet) in Japanese is not based on phonological codes. In brief, in Japanese, there are two writing systems, syllabic kana, which has two allographic forms hiragana and katakana (e.g., き and キ, both representing the syllable sound /ki/), and logographic kanji (e.g., 木, meaning “tree”, also pronounced /ki/). Importantly, kanji and kana are distinct writing systems and a kanji character homophonic with a kana cannot be considered an allograph, hence has no orthographic overlap. The experiment showed that priming of a single kana target by a homophonic kanji character (e.g., 木–き; 木–キ) was substantially smaller than the priming effect produced by an allograph kana prime (e.g., キ–き; き–キ), mirroring the present result that the orthographic priming effect produced by a cross-case identity prime (e.g., cult–CULT) is much greater than the phonological priming produced by a homophone prime (e.g., kult–CULT).

## Conclusion

In three experiments using words and pseudowords written in the Roman alphabet, within-script priming (Experiments 1 and 2 here and Experiment 3 of Kinoshita & Norris, 2009) the largest Bayes factor in favor of a phonological priming effect was 2 (Experiment 2). Lupker et al. (2015a) posed the question: Is there phonologically based priming in the same–different task? When, as in their experiments, the prime and target are presented in different orthographies the answer is yes. When, as in our experiments, they are presented in the same orthography, the answer is at best, in Jeffreys’ (1961) terms: “barely worth a mention.” Based on the arguments and the data presented here, there seems to be little reason to revise the assumption that data from masked priming tasks are telling us about the operation of early orthographic processes in letter and word recognition.

## Figures and Tables

**Table 1:** Mean Decision Latencies (Reaction Times in Ms), and Percent Error Rates (%E) in Experiment 1 (Word Referent/target)

Response and prime type	Example	RT	%E
Same trials (e.g., referent–cult, target–CULT)			
Identity	cult	451	3.5
PSH	kult	485	4.5
Control	nult	487	6.5
Different trials (e.g., referent–seed, target–CUST)			
Identity	cult	536	5.3
PSH	kult	532	4.8
Control	nult	538	3.0
*Note*. PSH = pseudohomophone.			

**Table 2 tbl2:** Mean Decision Latencies (Reaction Times in Ms), and Percent Error Rates (%E) in Experiment 2 (Pseudoword Referent/target)

Response and prime type	Example	RT	%E
Same trials (e.g., referent–cust, target–CUST)			
Identity	cust	451	6.1
PSH	kust	467	7.3
Control	nust	480	8.5
Different trials (e.g., referent–pibb, target–CUST)			
Identity	cust	513	4.4
PSH	kust	494	3.2
Control	nust	503	3.7

## Footnotes

1 Garcia-Orza and colleagues (2010, 2011) also identified the boundary condition: A string of complex visual objects (characters in “dingbat” fonts e.g., ) and pseudofonts do not produce these effects. (Although the authors’ interpretation is somewhat different) these results suggest that the referent string of “visual objects” needs to be simple and/or familiar to be easily codable and not exceed the capacity of visual short-term memory (for recent evidence regarding the role of object familiarity on the capacity of visual short term memory, see Xie & Zhang, 2017).

2 The overlap model is a model of perceptual identification and does not make quantitative predictions for masked priming effects.

3 Analogously, in lexical decision, the “word” decision may be based on the summed probabilities that the stimulus letter string matches a word in the lexicon (Norris, 2006) or “global lexical activation” (e.g., Grainger & Jacobs, 1996).

4 Schiller (2007) showed that this effect is due to phonological, not orthographic overlap (e.g., kapper–CONDOR < navel–CONDOR, but cijfer–CONDOR = navel–CONDOR; the primes and targets were all words in Dutch).

5 Following the present Experiment 1 and 2, Kinoshita et al. (2018) conducted a further experiment using Japanese kana and kanji characters. This experiment will be described in the General Discussion.

6 In the same–different task, masked priming effects are limited to the Same responses unless the prime is the same as the referent (the “zero-contingency” condition as used by Lupker et al., 2015: see Kinoshita & Kaplan, 2008, Experiment 3; Kinoshita & Norris, 2010). Readers are referred to Norris and Kinoshita (2008) and Kinoshita and Norris (2010) for a detailed explanation.

7 We hasten to add that Rastle and Brysbaert (2006) did not deny the role of phonology in lexical access; also, a recent review of the literature by Halderman, Ashby, and Perfetti (2012) pointed to “an early and integral role” of phonology in identifying words. Our point is that the orthographic priming effect is not due solely to phonological similarity; there is no contradiction between these views.

## List of Stimuli Used

### Critical Stimuli Used in Experiment 1

They are listed in the order: identity prime/pseudohomophone (PSH) prime/control prime/target (in uppercase letters)

cage/kage/fage/CAGE, cape/kape/mape/CAPE, card/kard/nard/CARD, care/kare/sare/CARE, cask/kask/zask/CASK, clip/klip/glip/CLIP, code/kode/sode/CODE, cold/kold/yold/COLD,

cord/kord/pord/CORD, corn/korn/sorn/CORN, gene/jene/fene/GENE, seam/ceam/heam/SEAM,

sect/cect/dect/SECT, self/celf /helf/SELF, sick/cick/bick/SICK, side/cide/lide/SIDE, class/klass/plass/CLASS, cliff/kliff/pliff/CLIFF, cycle/sycle /vycle/CYCLE, gypsy/jypsy/mypsy/GYPSY, carp/karp/farp/CARP, cave/kave/tave/CAVE, clap/klap/blap/CLAP, cool/kool/vool/COOL, core/kore/vore/CORE, cost/kost/fost/COST,

crop/krop/brop/CROP, cuff/kuff/vuff/CUFF, cult/kult/nult/CULT, gist/jist/nist/GIST,

seal/ceal/yeal/SEAL, seek/ceek/neek/SEEK, seem/ceem/veem/SEEM, seen/ceen/feen/SEEN,

send/cend/hend/SEND, sing/cing/hing/SING, cider/sider/bider/CIDER, clean/klean/slean/CLEAN, cream/kream/tream/CREAM, crest/krest/drest/CREST,

cake/kake/dake/CAKE, call/kall/jall/CALL, came/kame/vame/CAME, cane/kane/tane/CANE,

cart/kart/jart/CART, case/kase/dase/CASE, cast/kast /gast/CAST, cube/kube/dube/CUBE,

cure/kure/hure/CURE, curl/kurl/vurl/CURL, germ/jerm/nerm/GERM, kale/cale/rale/KALE,

seat/ceat/keat/SEAT, seed/ceed/veed/SEED, sink/cink/hink/SINK, size/cize/nize/SIZE,

coast/koast/noast/COAST, giant/jiant/fiant/GIANT, kayak/cayak/mayak/KAYAK, sight/cight/vight/SIGHT

### Critical Stimuli Used in Experiment 2

They are listed in the order: identity prime/PSH prime/control prime/target (in uppercase letters)

calp/kalp/halp/CALP, ceck/seck/weck/CECK, celp/selp/melp/CELP, cert/sert/rert/CERT, cirt/sirt/lirt/CIRT, colp/kolp/rolp/COLP, coom/koom/poom/COOM, coph/koph/boph/COPH, corg/korg/forg/CORG, corz/korz/morz/CORZ, cust/kust/nust/CUST, jick/gick/yick/JICK,

seld/celd/reld/SELD, sepp/cepp/mepp/SEPP, siff/ciff/diff/SIFF, sirk/cirk/hirk/SIRK,

caple/kaple/waple/CAPLE, civer/siver/tiver/CIVER, koost/coost/voost/KOOST,

jitle/gitle/hitle/JITLE, carb/karb/varb/CARB, cegg/segg/degg/CEGG, cerk/serk/nerk/CERK,

cipt/sipt/hipt/CIPT, cive/sive/mive/CIVE, cont/kont/jont/CONT, coor/koor/loor/COOR,

corb/korb/yorb /CORB, corp/korp/vorp/CORP, crot/krot/brot/CROT, cynt/synt/rynt/CYNT,

jiph/giph/viph/JIPH, selt/celt/jelt/SELT, serl/cerl/nerl/SERL, simm/cimm/vim/SIMM,

sith/cith/nith/SITH, citch/sitch/fitch/CITCH, clist/klist/plist/CLIST, cralp/kralp/tralp/CRALP,

setch/cetch/metch/SETCH, carg/karg/yarg/CARG, celk/selk/velk/CELK, cerp/serp/nerp/CERP,

cirp/sirp/girp/CIRP, clor/klor/slor/CLOR, coob/koob/foob/COOB, coot/koot/voot/COOT, corf/korf/morf /CORF, cors/kors/jors/CORS, culk/kulk/julk/CULK, jert/gert/fert/JERT,

sein/cein/zein/SEIN, senf/cenf/genf/SENF, sest/cest yest/SEST, sint/cint/fint/SINT

symp/cymp/bymp/SYMP, citle/sitle/ritle/CITLE, cotle/kotle/lotle/COTLE, crodd/krodd/brodd/CRODD, siple/ciple/viple/SIPLE
